# Compliance with U.S. Government Nutrition Advice and Concurrent Obesity Trends Using Nurses' Health Study Data, 1980–2011

**DOI:** 10.1016/j.tjnut.2023.11.010

**Published:** 2023-11-19

**Authors:** Evan K Cohen, Dennis Bier, Matthew Martinez

**Affiliations:** 1 The Brattle Group, Boston, MA, U.S.; 2 Baylor College of Medicine, Houston, TX, U.S.; 3 George Washington University, Washington DC, U.S.

**Keywords:** Nurses’ Health Study, US Dietary Guidelines, obesity, fat, saturated fat, cholesterol, BMI

## Abstract

**Background:**

Beginning in 1977, the U.S. Government began formally issuing dietary advice, a main objective of which was to reduce and prevent the prevalence of obesity in the American population. Concurrently, the Harvard School of Public Health began conducting dietary intake surveys and collecting body mass index (BMI) (kg/m^2^) data on female nurses in the Nurses’ Health Study I (NHSI) and II (NHSII).

**Objectives:**

We aimed to assess whether compliance with the nutrition guidance from the U.S. Government to restrict dietary intake regarding total fat, saturated fat, and cholesterol was meaningfully associated with the prevalence of obesity.

**Methods:**

We analyzed nutrition survey data from 1980 to 2011, grouping the sample into “compliers,” those who complied with guidance on the intake of total fat, saturated fat, and cholesterol, and “noncompliers,” those who did not. We then compared the means, medians, and distributions of BMI for compliers and noncompliers over the period for both the full survey population and an age-controlled sample. Finally, we plotted raw NHS data to examine respondents’ Fat Proportion intake of energy and concurrent BMI.

**Results:**

The mean and median BMI for both compliers and noncompliers increased throughout the sample period, and the BMI distributions shifted toward obese and severely obese overall and for an age-controlled subset compared with the 1980 NHSI and 1990 NHSII baselines. Compliers had slightly lower mean BMI increases than noncompliers but saw a relatively higher increase in the growth of the prevalence of those with BMI >30. We also found no linear relationship between Fat Proportion of energy intake and concurrent BMI.

**Conclusions:**

Guidance from the U.S. Government to limit fat, saturated fat, and cholesterol consumption was widely adopted by American female nurses during the study period. Our results show that compliance with this guidance had little if any effect in mitigating population-wide BMI increases during our study period.

## Introduction

In 1977, the U.S. Senate Select Committee on Nutrition and Human Needs issued 2 editions of a report entitled “Dietary Goals for the U.S.” (the “1977 Senate Reports”), beginning an era of the U.S. Government offering formal guidance on what to eat [1,2]. Spurred by the 1977 Senate Reports, the first Dietary Guidelines for Americans (“USDGAs”) were rolled out in 1980 and were updated by statute every 5 y thereafter [[3], [4], [5], [6], [7], [8], [9]]. The overarching principle of the nutrition advice offered in the 1977 Senate Reports and the USDGAs (collectively “ U.S. Government Nutrition Advice”) was to address the link between diet and disease and offer guidance on what people should eat to “stay healthy” and avoid or mitigate chronic disease. To do so, the U.S. Government Nutrition Advice included recommendations for macronutrients, salt, sugar, and cholesterol consumption, among others. One hope was that U.S. Government Nutrition Advice would mitigate obesity, as it was considered a major risk factor for comorbidities such as heart disease, cancer, stroke, and diabetes. Mitigation of the prevalence of obesity was generally focused on energy balance, with recommendations given to eat less, eat fewer nutrient-dense foods, and exercise more. Thus, the solution underlying U.S. Government Nutrition Advice was focused on a shift from fat, “the most concentrated energy source,” [2] to bulkier, less energy-dense, complex carbohydrate-rich foods [[1], [2], [3], [4], [5], [6], [7], [8], [9]].

Approximately concurrent with the advent of U.S. Government Nutrition Advice, the Nurses’ Health Study (NHS) I began collecting data on 121,700 female nurses in 1976, and NHSII began collecting data on a different cohort of 116,686 female nurses in 1989. The first set of data for NHSI was published for 1980 responses, and the first set of data for NHSII was published for 1991 responses. To our knowledge, these are among the most comprehensive longitudinal health data ever collected. Participants were tracked periodically over decades, completing detailed questionnaires about medical, lifestyle, and health-related information. The data are unique in the sheer number of participants, the breadth of data collected, the length of time participants were tracked, and the range of analyses published using these underlying data.

Access to the NHS data has enabled us to track compliance with several of the quantitative metrics from the U.S. Government Nutrition Advice, allowing us to examine whether the respondents in our sample generally adhered to the U.S. Government Nutrition Advice offered from 1977 to 2011. We report compliance with the U.S. Government Nutrition Advice to limit Fat Proportion, Saturated Fat Proportion, and cholesterol intake as a proxy for general adherence to the U.S. Government Nutrition Advice. Our analysis of the Studies’ data examined the association between compliance with the U.S. Government Nutrition Advice and BMI, both within and across Study years, considering both the overall Studies’ cohorts and an age-controlled sample.

## Methods

### NHS data

In this study, we utilized NHS data to analyze long-term macronutrient consumption and BMI trends in the U.S.. NHS nutrition survey data were collected every 4 y beginning in 1980 for NHSI (except for a 2 y gap from 1984 to 1986), and 1991 for NHSII. Our analysis included all reported data for NHSI through 2010, and 2011 for NHSII. NHSI’s initial criteria for inclusion were married female nurses between the ages of 30 y and 55 y in 1976, living in the 11 most populous states. NHSII included female nurses between the ages of 25 y and 42 y in 1989, living in the 14 most populous states. As a result of the inclusion criteria, the data’s demographic diversity (race, gender, ethnicity, income, and education level) is limited. In aggregate, only 5.4% of the eligible participants in the Studies who met the criteria for inclusion in our analysis identify as non-White. The Studies relied on self-reporting for physical attributes such as height and weight [10] and on nutritional surveys asking participants to estimate their consumption of various types of food over the previous year [11]. Nutrition information for the Studies was collected through a food recall survey, for which researchers have previously developed a methodology for converting into detailed consumption data [12,13]. We build off the work of these researchers, accepting and using their methodology for this conversion. As such, our reported nutrition information should be comparable to all prior work that has utilized this conversion methodology from the data in the Studies.

We restricted the data from the Studies to create our samples for the analysis. First, we limited the samples to only include respondents between the ages of 18 and 64 y in any given Study year; we excluded participants >65 y of age due to “possible confounding due to age-related loss of lean muscle mass” [14]. Second, we excluded pregnant and lactating women in NHSI in the years they reported being pregnant or lactating. We could not make the same adjustment to our NHSII sample because it did not include the required variables to make this adjustment. Finally, we excluded women with missing data to calculate BMI in any given year. After making these adjustments, our samples included 91,472 women in NHSI in 1980 (75.2% of the total sample and 98.9% of initial food survey respondents) and 92,562 women in NHSII in 1991 (79.3%; 97.0%, respectively).

### U.S. Government Nutrition Advice, 1977–2010

We compiled a dataset to record U.S. Government Nutrition Advice for Fat Proportion, Saturated Fat Proportion, and cholesterol consumption levels from 1977 to 2010. As discussed below, although other advice on macronutrient, submacronutrient, salt, and food groups were included over the years, these were the only 3 quantitative advisory measures that were relatively consistent throughout the study period. We used these metrics as a proxy for overall compliance with U.S. Government Nutrition Advice.

### Caloric intake and compliance data

We computed the mean Fat Proportion, Saturated Fat Proportion, and cholesterol in the diet of individual female nurses in the NHSI and NHSII surveys over the corresponding sample periods. To do so, we first transformed our underlying data in such a way that fat and saturated fat consumption were defined in terms of caloric intake and cholesterol intake was defined in terms of mg/d. We converted the data to be reported in terms of caloric intake by multiplying the following variables using the ensuing conversion rates: carbohydrates (4 kcal/g), protein (4 kcal/g), fat (9 kcal/g), and alcohol (7 kcal/g) [2].We then calculated Fat Proportion by dividing our measure of calculated energy from fat (i.e., 9 times grams of fat) by total mean individual energy intake. We calculated Saturated Fat Proportion in the same manner.

Additionally, in any given year, we defined a Study participant as being overall compliant with the U.S. Government Nutrition Advice if the individual met the criteria for following each of the 3 individual advisories by satisfying these conditions: *1)* annual mean Fat Proportion <30%/d of total energy intake; *2)* annual mean Saturated Fat Proportion <10%/d of total energy intake; and *3)* annual mean cholesterol consumption <300 mg/d. We labeled those who complied with the individual standards as compliers or complying with those individual recommendations and those complying with all 3 standards simultaneously in any given survey year as overall compliers or overall complying.

### Compliance and BMI outcomes

First, to understand how BMIs have generally changed over the sample period, we estimated and plotted the annual mean and median BMIs for female nurses in the NHSI and NHSII surveys separately over the sample periods for each. Next, to view how the distributions of BMIs in these data have changed over time, we used a Gaussian kernel density estimator to estimate the distributions of BMIs for nurses included in the analysis in 1980 (using data from NHSI) and in 2010/2011 (using data from NHSI and NHSII). To control for the effects of aging, we used the same procedure to estimate the distributions of BMIs for women between 55 and 64 y of age in 1980 and in 2010/2011. We chose to analyze the oldest range in our sample (55–64-y olds) because it allowed us to include the largest number of observations across the earliest and latest years covered by this analysis, 1980 and 2010/2011; in fact, for NHSI, because of the age range for initial inclusion and the 30-y span of the analysis, this is the only age range with participants in both 1980 and 2010. Note that for ease of interpretation, we limited the axes of our figures (but not the underlying analysis) to show only BMIs between 14 and 55.

We then calculated the share of respondents in our samples that fell into different BMI categories in 1980 compared with 2010/2011 and calculated the sample growth rate from 1980 to 2010/2011 in each BMI category. We used the U.S. Centers for Disease Control and Prevention BMI categories to classify the respondents: <18.5—underweight, 18.5 < 25—normal, 25 < 30—overweight, 30 < 40—obese, and ≥ 40—severely obese [15].

Next, we again used a Gaussian kernel density estimator to estimate the distributions of BMI for 55–64-y-old female nurses for those classified as compliers in 1980 and 2010/2011, separately, compared with those classified as noncompliers in 1980 and in 2010/2011, separately. We also estimated mean and median BMI for compliers and noncompliers in 1980 and 2010/2011. We then estimated a 2-way ANOVA model to statistically test the relationships between time period (1980 compared with 2010/2011) and compliance on mean BMI. Further, we calculated the proportion of female nurses in NHSI and NHSII aged 55–64 y who fell into each BMI category (by overall compliance status) in 1980 and 2010/2011.

Finally, because the Second Edition of the 1977 Senate Report stated that reducing total fat consumption in particular was key to the equation of changing the dietary energy imbalance to control obesity by limiting energy-dense fats in favor of bulkier, less energy-dense complex carbohydrates, we plotted BMI and mean annual relative consumption of fat energy for each respondent in our sample for each survey year. Additionally, we calculated the interquintile range (IQR) of Fat Proportion for normal BMI levels across the survey years, which showed the range of the middle 80% of the distribution in each year.

## Results

### U.S. Government Nutrition Advice

Table 1 is a summary of the U.S. Government Nutrition Advice on Fat Proportion, Saturated Fat Proportion, and mean daily cholesterol intake from 1977 to 2010.

**TABLE 1:** U.S. Government Nutrition Advice on Fat Proportion, Saturated Fat Proportion, and cholesterol intakes

Year	Fat Proportion	Saturated Fat Proportion	Cholesterol
1977	<30% energy	<10% energy	<300 mg/d
1980 and 1985	Avoid too much fat, saturated fat, and cholesterol.		
1990	<30% energy	<10% energy	Eat less cholesterol
1995	<30% energy	<10% energy	<300 mg/d
2000	<30% energy	<10% energy	<300 mg/d
2005	20%–35% energy	<10% energy	<300 mg/d
2010	20%–35% energy	<10% energy	<300 mg/d

### NHS data

Table 2 shows that as participant’s age or drop out of the Studies, the number of potential participants in any given year drops and gets older. The last 2 columns in Table 2 show the mean age of the participants in the Studies and of the participants included in our samples. For NHSII, since the data span a 20-y period and the participants were aged between 25 y and 42 y in 1989 when the survey began, no participants were excluded from our sample because of their aging beyond our age criteria. However, since NHSI began collecting data in 1976, and the minimum age for inclusion in the survey was 30 y in 1976, most participants did not meet our age inclusion criteria by 2010.

**TABLE 2:** NHS sample

	Number of respondents		Mean age of respondents (y)	
NHSI				
Year	Considered	Included	Considered	Included
1980	92,468	91,472	46.4	46.4
1984	81,757	77,601	50.5	50.5
1986	73,666	70,411	52.7	52.4
1990	78,884	63,738	56.5	54.4
1994	84,650	56,321	60.3	56.3
1998	82,647	42,347	64.2	58.3
2002	78,191	25,201	67.9	60.2
2006	66,337	12,723	71.4	62.0
2010	63,602	2,586	74.7	63.7

NHSII				

Year	Considered	Included	Considered	Included

1991	95,452	92,562	36.1	36.1
1995	85,101	81,645	40.2	40.2
1999	85,122	81,097	44.3	44.2
2003	72,165	68,672	48.4	48.3
2007	74,072	71,286	52.5	52.4
2011	65,629	63,006	56.5	56.5

Respondents were considered for this analysis if they reported caloric intake in a given year. Respondents were included in the analysis if they had nonmissing BMI and were under the age of 65 y in a given year. The last 2 columns show the mean age of considered and included respondents in a given year. NHS, Nurses’ Health Study.

### Consumption and compliance trends

#### Mean share of energy in diet from total fat and saturated fat, and total cholesterol intake

**FIGURE 1:**
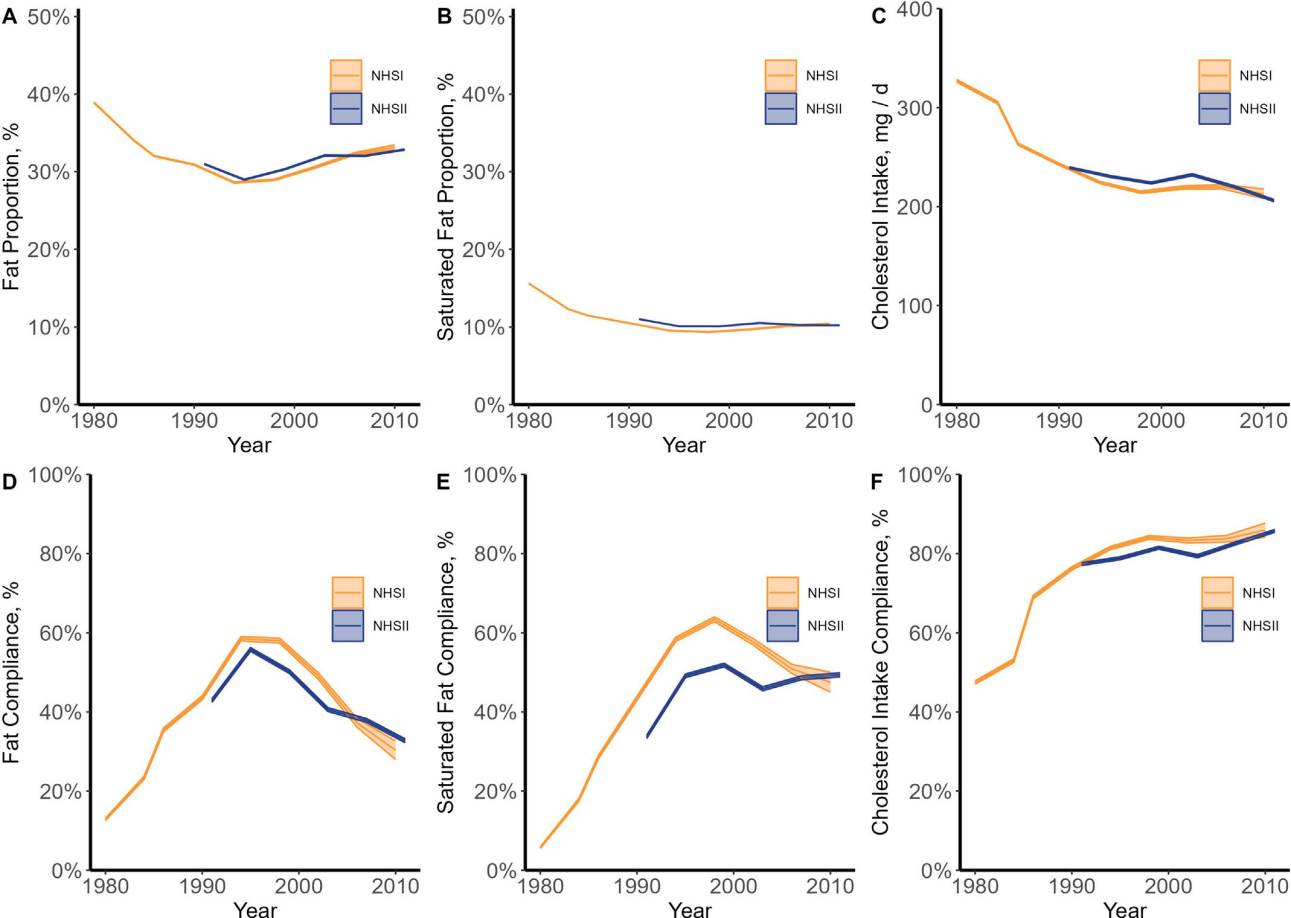
Fat Proportion (A) and Saturated Fat Proportion (B) intakes, absolute cholesterol intake (C), and rates of compliance with each specific U.S. Government Nutrition Advice (D-F) by female nurses in NHSI (qualified initial cohort *n* = 91,472) and NHSII (qualified initial cohort *n* = 92,562) from 1980 to 2011. Lines are mean point estimates, and shaded areas represent 99% confidence intervals. NHS, Nurses’ Health Study.

Figures 1A, B, and C linearly plot mean Fat Proportion, Saturated Fat Proportion, and mean total cholesterol consumption, respectively, for each food survey year from 1980 to 2011 for NHSI and NHSII. Figures 1D, E, and F linearly plot the proportion of respondents who complied with the individual U.S. Government Nutrition Advice on Fat Proportion, Saturated Fat Proportion, and cholesterol consumption, respectively, for each food survey year from 1980 to 2011 for NHSI and NHSII. All panels in Figure 1 include the 99% CIs (confidence intervals) for our point estimates (shown as the shaded areas).

*Fat Proportion and Fat Proportion compliance.*Figure 1A shows a decrease in mean Fat Proportion from the baseline of the first observations in 1980, where NHSI showed mean Fat Proportion at 39% of total energy intake. Fat Proportion fell rapidly, and the 30% recommendation benchmark from the U.S. Government Nutrition Advice was achieved on average in the mid- and late 1990s in both NHSI (1994 and 1998) and NHSII (1995), before mean Fat Proportion rose slightly in both Studies, although not approaching the level from 1980.

Figure 1D shows the share of each Study population that complied with the U.S. Government Nutrition Advice recommendations to limit Fat Proportion to 30% of total energy intake in any given year. In particular, it shows that the compliance rate grew from 13% in 1980 to >50% in the 1990’s, then fell to slightly >30% over the final decade of our study.

*Saturated Fat Proportion and Saturated Fat Proportion compliance.*Figure 1B shows that mean Saturated Fat Proportion in 1980 was 16% of energy intake but rapidly declined to meet the 10% recommendation benchmark on average by the late 1990s. Mean Saturated Fat Proportion reached its minimum in the late 1990s, plateaued in the 2000s, and rising slightly in the final decade of this data.

Figure 1E shows that the share of Study participants who complied with the U.S. Government Nutrition Advice to consume <10% of total energy intake from saturated fat was 6% in 1980, peaked at >50% in the late 1990’s, and finished at ∼50% at the end of the Study periods in 2010 and 2011.

*Dietary cholesterol and dietary cholesterol compliance.*Figure 1C illustrates that compliance with U.S. Government Nutrition Advice on cholesterol was marked by quick and consistent adoption of the guidelines throughout the Study period. The data show that American female nurses largely followed the advice to consume 300 mg/d of cholesterol. Mean cholesterol consumption in 1980 was only slightly above the U.S. Government Nutrition Advice at a mean of 327 mg/d, but by 1986, mean cholesterol consumption had fallen to 263 mg/d and generally fell every year thereafter for both Studies, hovering at just >200 mg/d at the end of the Study periods in 2010 and 2011.

Figure 1F shows that the mean compliance rate for cholesterol intake started out at just <50% in 1980 but grew steadily and consistently throughout the Study period, peaking at >80% at the end of the Study period in 2010 and 2011. As such, the data show that the U.S. Government Nutrition Advice on dietary cholesterol consumption has largely been adopted.

#### Overall compliance

**FIGURE 2:**
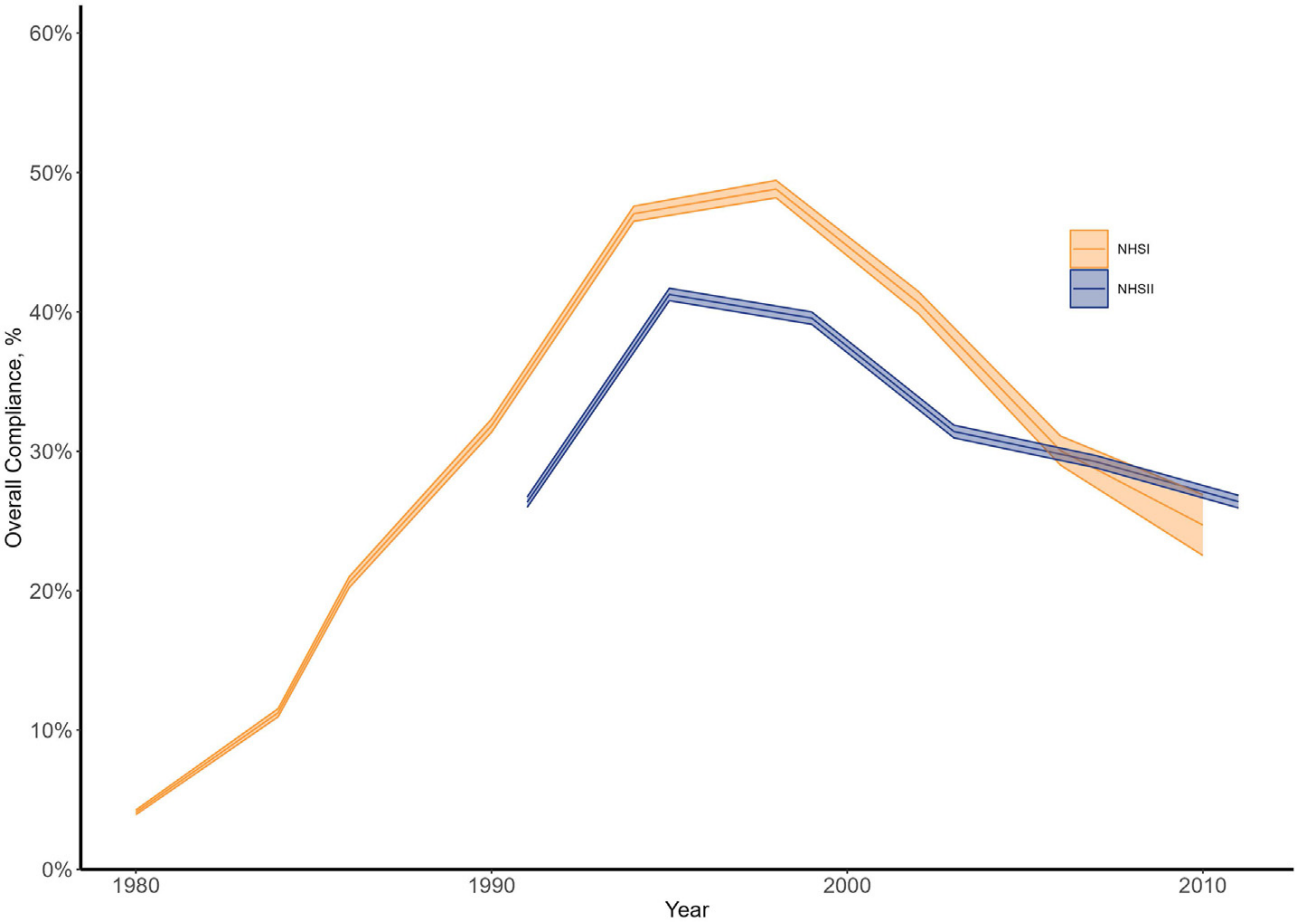
Overall compliance with U.S. Government Nutrition Advice on simultaneous compliance for Fat Proportion, Saturated Fat Proportion, and cholesterol intakes by female nurses in NHSI (qualified initial cohort *n* = 91,472) and NHSII (qualified initial cohort *n* = 92,562) from 1980 to 2011. Lines are mean point estimates, and shaded areas represent 99% confidence intervals. NHS, Nurses’ Health Study.

Figure 2 shows overall compliance with all 3 U.S. Government Nutrition Advice recommendations simultaneously: Fat Proportion <30%, Saturated Fat Proportion <10%, and total cholesterol <300mg/d. In 1980, only 4% of the NHSI cohort demonstrated overall compliance with all 3 guidelines; in 1998, 49% of the NHSI cohort overall complied. For NHSII, which began collecting data in 1990, the compliance trends were similar to that of NHSI for much of the period but slightly lower until the mid-2000s. Note that we have not attempted to control for age in this figure, and the considered NHSI population was substantially older than the considered NHSII population at any given point in time (as shown in Table 2).

### BMI trends

#### Mean and median BMI trends

**FIGURE 3:**
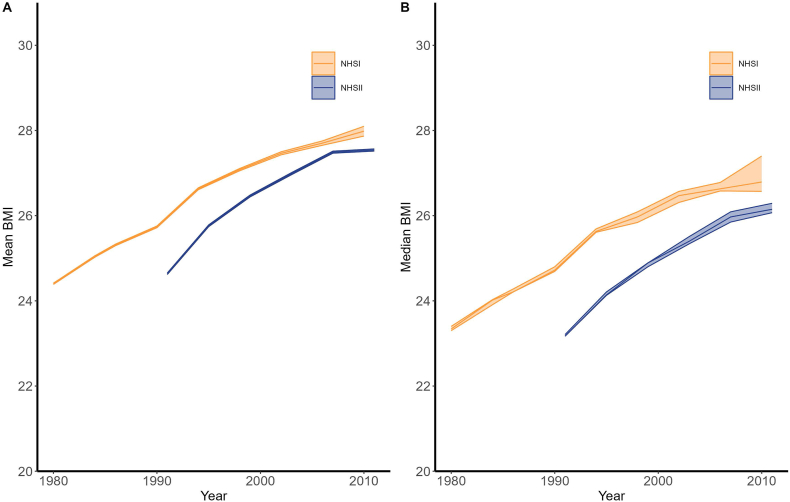
Mean (A) and median (B) BMI over time for female nurses in NHSI (qualified initial cohort *n* = 91,472) and NHSII (qualified initial cohort *n* = 92,562) from 1980 to 2011. Lines are mean (A) and median (B) point estimates, and shaded areas represent 99% confidence intervals. NHS, Nurses’ Health Study.

Figure 3 shows that the female nurses in these Studies underwent a steep growth in mean and median BMI throughout the Study period. Starting mean and median BMIs in 1980 for the NHSI cohort were both around 24 but grew consistently throughout the period, peaking at a mean of ∼28 and a median of ∼ 27 in 2010. For NHSII, which began collecting data in 1990, the growth in mean and median BMIs over the sample period was quite similar; however, the values for NHSII were below that of NHSI in every year. Again, we have not attempted to control for age in this figure; thus, the disparity between the 2 results in any given year could be explained by the age disparity between the 2 cohorts.

#### BMI distribution trends for 55–64-y-old women

**FIGURE 4:**
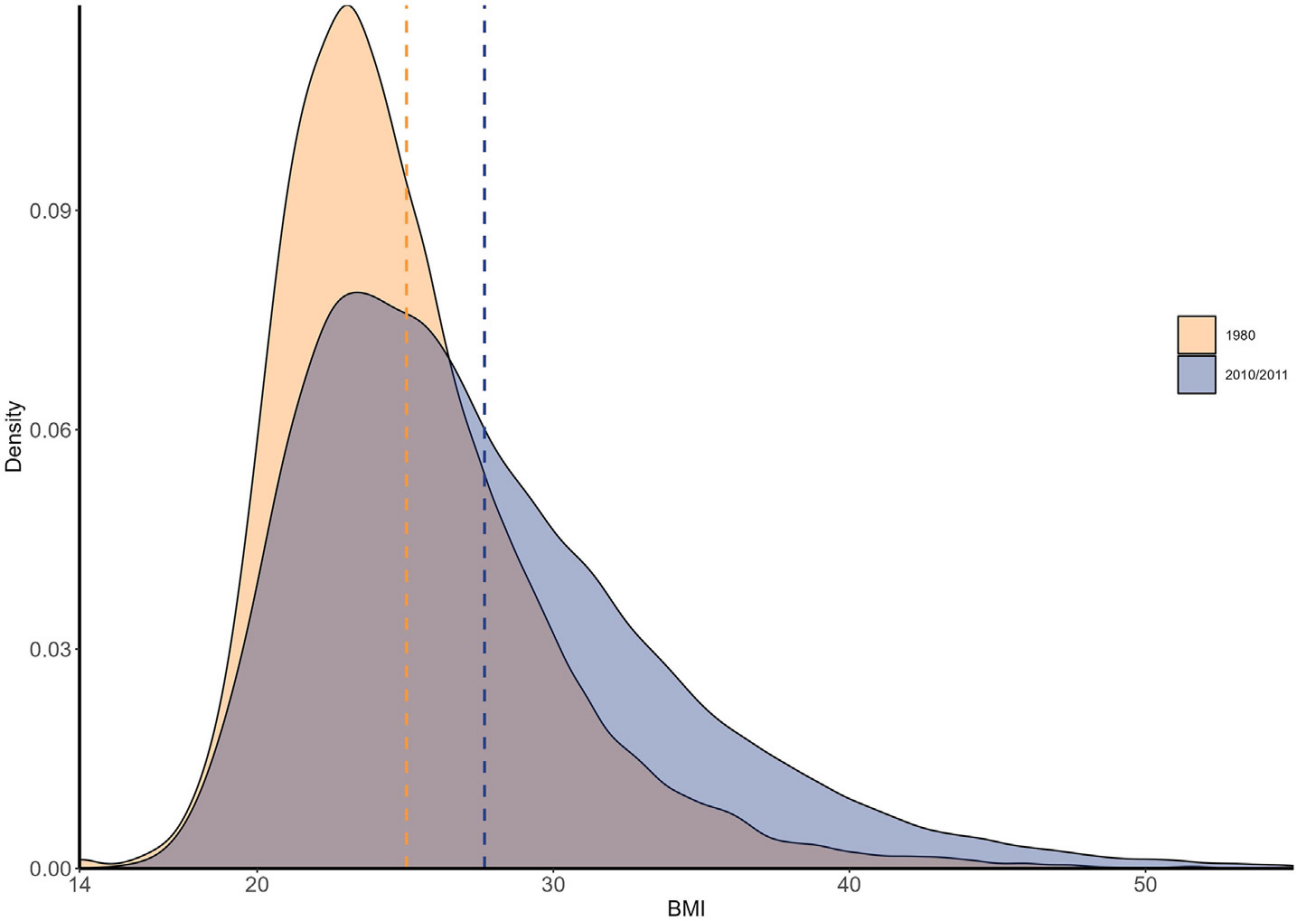
BMI distribution of 55–64-y female nurses in 1980 (NHSI, *n* = 91,472) and 2010/2011 (NHSI and NHSII combined, *n* = 65,592) using Gaussian kernel density estimation. Dashed lines are the means for the corresponding color time point. Figure shows BMI between 14 and 55, although all reported BMI values were included for density estimation. NHS, Nurses’ Health Study.

Figure 4 shows a flattening and right-skewing of the 2010/2011 distribution of BMI for American female nurses aged 55–64 y compared with the distribution of BMI in 1980. This shows that not only had the mean and median BMIs of female nurses increased over this period, but a larger share of female nurses became overweight, obese, or severely obese.

Table 3 shows the share of 55–64-y-old women in each BMI category in 1980 and 2010/11. The share of women classified as underweight and normal weight dropped over the 30 y period by 29% and 32%, respectively. Those classified as overweight rose only 7%. In contrast, those classified as obese grew 119%, and those classified as severely obese grew 356%, from just 1.0% of the women in 1980 to 4.7% in 2010/11.

**TABLE 3:** Mean, median, and share of 55–64-y-old female nurses by BMI category in 1980 and 2010/11

		BMI		Share of sample by BMI category				
	Respondents, *n*	Mean	Median	Underweight	Normal	Overweight	Obese	Severely obese
				BMI <18.5	18.5≤ BMI < 25	25 ≤ BMI <30	30 ≤BMI < 40	BMI ≥40
Year								
1980	15,383	25.04	24.14	1.7%	57.0%	29.0%	11.2%	1.0%
2010/2011	44,034	27.68	26.45	1.2%	38.5%	31.0%	24.6%	4.7%
Increase	–	11%	10%	−29%	−32%	7%	119%	356%

Total number of respondents: 1980 (*n* = 15,383); 2010/11 (*n* = 44,034).

### Overall compliance with U.S. Government Nutrition Advice and BMI distribution changes for 55–64-y-old female nurses

**FIGURE 5:**
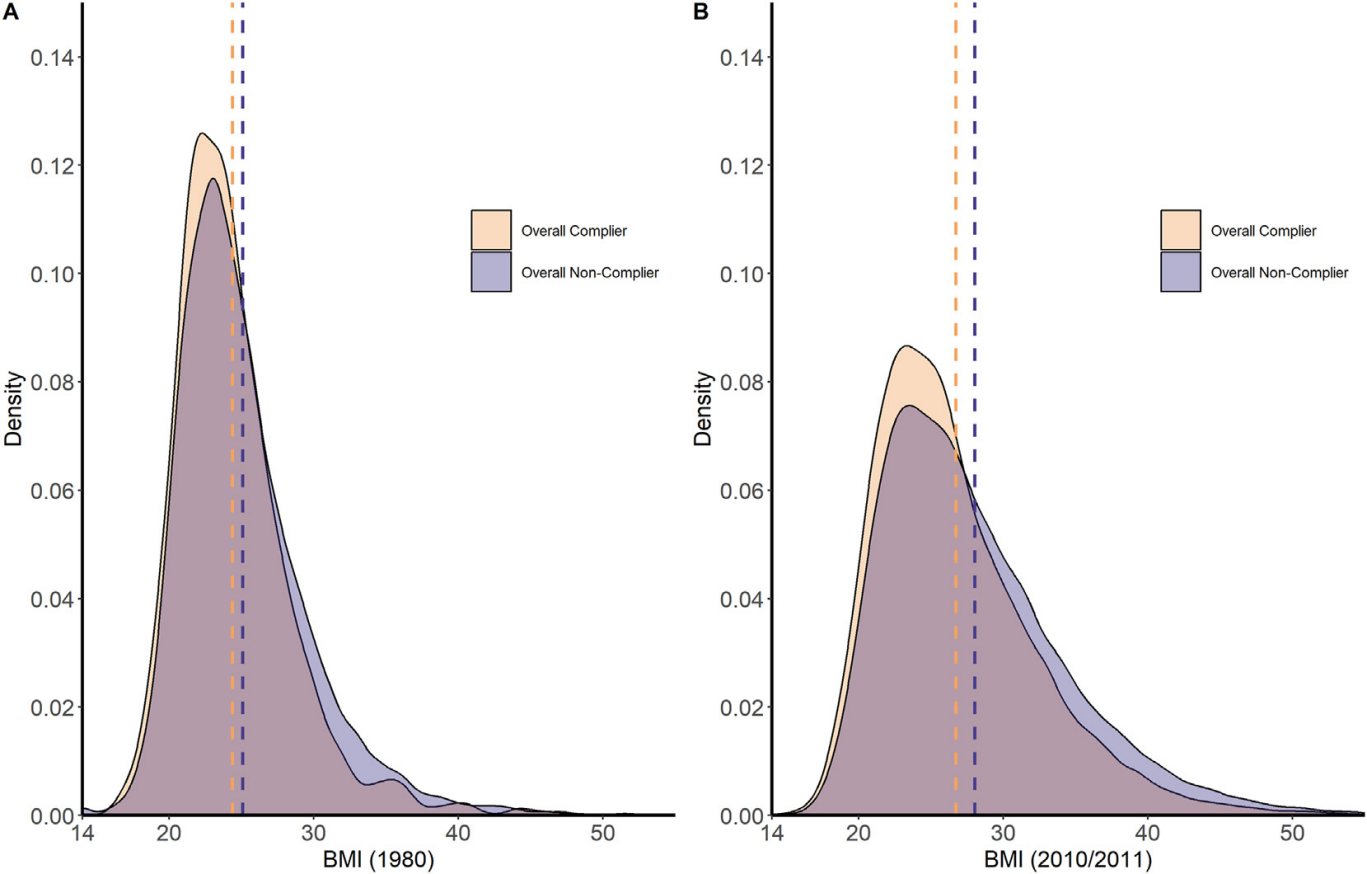
Distribution of 55–64-y-old female nurses’ BMI in 1980 (NHSI, *n* = 91,472) (A) and 2010/2011 (NHSI and NHSII combined, *n* = 65,592) (B) by overall compliance with U.S. Government Nutrition Advice using Gaussian kernel density estimation. Dashed lines are the means for the corresponding compliance color. Figure shows BMI between 14 and 55, although all reported BMI values were included for density estimation. NHS, Nurses’ Health Study.

Figure 5 shows that the larger trends in the changes in distribution of BMI from 1980 to 2010/2011, shown in Figure 4, persist for both groups when the data are bifurcated between overall compliers and overall noncompliers. Both age-controlled groups experienced a similar flattening and right-skewing of BMI distributions in 2010/2011 compared with 1980. As shown in more detail in Table 4, compliers had lower mean BMI in both 1980 and 2010/11 compared with noncompliers.

**TABLE 4:** Mean and median BMI bifurcated by overall compliance status of 55–64-y-old female nurses, 1980 and 2010/11

	Overall complier	Overall noncomplier	Difference
Respondents			
1980	922	14,461	–
2010/2011	11,786	32,248	–
Mean BMI			
1980	24.37	25.09	0.72
2010/2011	26.72	28.03	1.30
Increase (%)	9.7	11.7	
Median BMI			
1980	23.69	24.21	0.52
2010/2011	25.61	26.63	1.02
Increase (%)	8.1	10.0	

Respondents in 1980 (*n* = 922 for compliers and *n* = 14,461 for noncompliers) and 2010/11 (*n* = 11,786 for compliers, *n* = 32,248 for noncompliers).

Table 4 shows that mean and median BMIs of qualified respondents classified as overall compliers were slightly lower than those classified as overall noncompliers in both 1980 and 2010/2011. In 1980, the mean BMI of overall noncompliers was 25.09, 0.72 units higher than the mean BMI of overall compliers of 24.37; in 2010/2011, the mean BMI of noncompliers was 28.03, 1.30 units higher than the mean BMI of compliers of 26.72. In 1980, the median BMI of noncompliers was 24.21, 0.52 units higher than the median BMI of compliers of 23.69; in 2010/2011, the median BMI of noncompliers was 26.63, 1.02 units higher than the median BMI of compliers of 25.61.

Notably, mean and median BMIs increased from 1980 to 2010/2011 for both overall compliers and overall noncompliers. Mean BMI for overall compliers increased by 9.7% over the sample period, and mean BMI for overall noncompliers increased by 11.7% over the sample period. Said differently, the increase in mean BMI for overall noncompliers was ∼21% greater than the increase in mean BMI for overall compliers, but both groups’ mean BMI increased by > 2.0 BMI units over the period.

Supplemental Table 1 shows the results from our 2-way ANOVA model to statistically test the relationships between time period (1980 compared with 2010/2011) and overall compliance on BMI. The result of the test shows that overall compliers experienced BMI growth over time that was statistically significantly different from overall noncompliers.

Table 5 bifurcates the analysis in Table 3 by overall compliance status, showing the change in BMI categories for overall compliers and overall noncompliers over the sample period. Overall compliers and overall noncompliers saw similar changes between 1980 and 2010/2011 in the share of respondents classified as underweight, normal weight, and overweight. However, overall, compliers saw a 216% increase in the share of respondents classified as obese over the 30-year period, whereas overall, noncompliers experienced a relatively smaller increase of 126%. The story changes for those classified as severely obese: the increase over the period for overall compliers was 227%, but for overall noncompliers, the increase was almost double at 416%.

**TABLE 5:** BMI categories for 55–64-y-old NHSI and NHSII respondents by overall compliance status

		Underweight (%)	Normal (%)	Overweight (%)	Obese (%)	Severely obese (%)
Year	Respondents	BMI < 18.5	18.5 ≤ BMI < 25	25 ≤ BMI <30	30 ≤ BM <40	BMI ≤ 40
1980						
Overall complier	922	2.1	63.4	27.1	6.5	0.9
Overall noncomplier	14,461	1.7	56.6	29.2	11.5	1.0
2010/2011						
Overall complier	11,786	1.5	43.8	31.2	20.6	2.8
Overall noncomplier	32,248	1.1	36.6	30.9	26.1	5.3
Change						
Overall complier	–	-25.1	-30.9	15.2	216.0	226.6
Overall noncomplier	–	-35.2	-35.3	5.9	126.4	416.4

Respondents by BMI category by overall compliance status in 1980 (*n* = 922 for overall compliers, *n* = 14,461 for overall noncompliers) and 2010/11 (*n* = 11,786 for overall compliers, *n* = 32,248 for overall noncompliers); NHS, Nurses’ Health Study.

### BMI and Fat Proportion compliance

**FIGURE 6:**
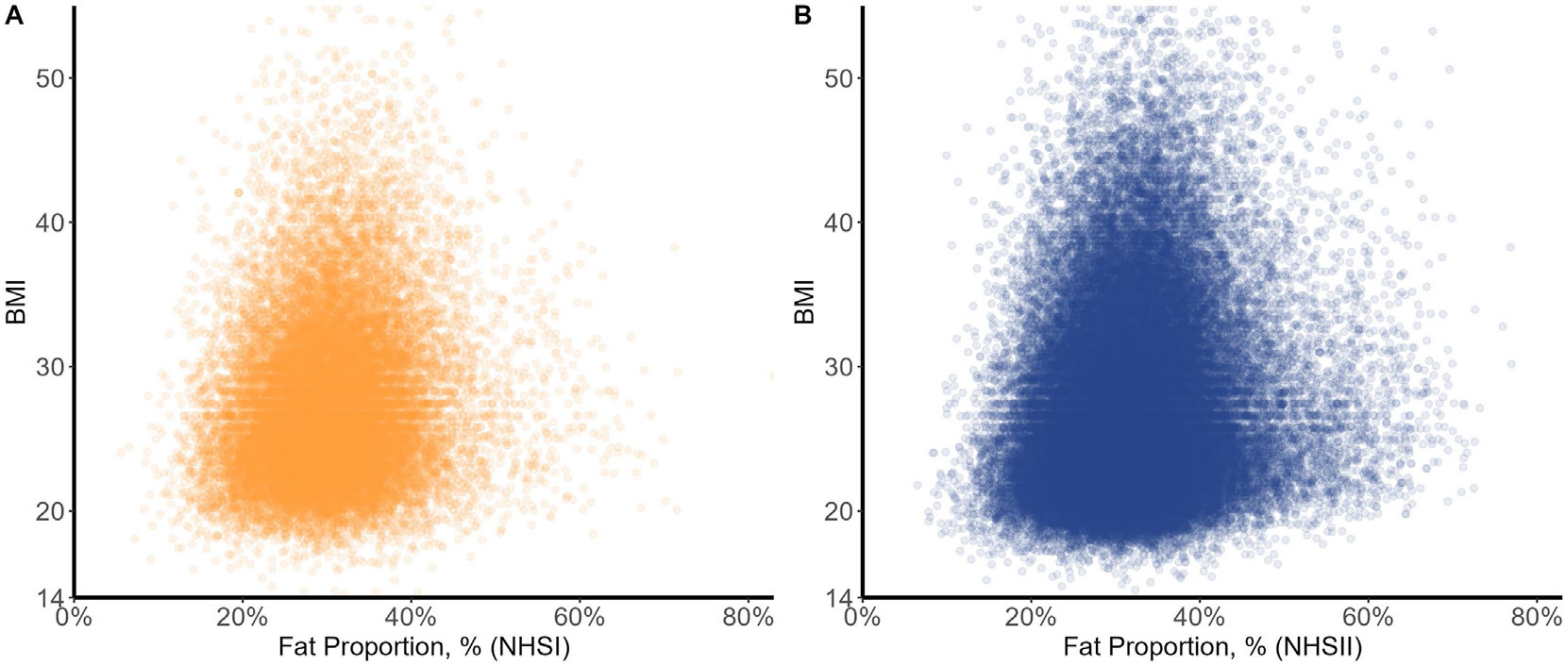
Female nurses’ BMI compared with Fat Proportion for 2002 NHSI (*n* = 25,152) (A) and 2003 NHSII (*n* = 68,530) (B). Only BMI values between 14 and 55 are shown—for 2002, this led to 49 excluded data points from the plot, and for 2003, this led to 142 excluded data points from the plot. NHS, Nurses’ Health Study.

Figure 6 shows Fat Proportion and corresponding BMI for each respondent in our NHSI and NHSII samples, respectively, in an exemplar year, 2002/03. We include corresponding figures for all other years in Supplemental Figure 1 and note that they present similar findings to Figure 6. As total fat consumption and total caloric consumption are the 2 components of the computation for Fat Proportion, we also present the individual figures by year for each component compared with BMI in Supplemental Figure 2 and Supplemental Figure 3.

Table 6 shows the first and fourth quintiles of Fat Proportion for nurses with normal BMI in any given year. The minimum range is 9.2%; in all years, the Quintile 4 values for respondents with normal BMI were above the U.S. Government Nutrition Advice to limit Fat Proportion to 30%.

**TABLE 6:** IQR of Fat Proportion for nurses with normal BMI values (18.5 ≤ BMI < 25) by Study and year

Year	Respondents	Quintile 1	Quintile 4	IQR
NHSI				
1980	58,361	32.3%	45.3%	13.0%
1984	45,096	29.0%	38.4%	9.3%
1986	39,045	27.0%	36.3%	9.2%
1990	32,846	25.3%	35.1%	9.8%
1994	24,822	22.2%	33.4%	11.2%
1998	17,044	22.3%	33.5%	11.2%
2002	9500	23.8%	35.2%	11.4%
2006	4600	26.1%	36.9%	10.8%
2010	879	27.5%	37.6%	10.1%
NHSII				
1991	58,159	25.9%	35.1%	9.2%
1995	44,639	22.9%	33.7%	10.8%
1999	40,363	24.1%	34.6%	10.5%
2003	31,469	25.5%	36.5%	11.0%
2007	29,462	26.5%	36.4%	9.9%
2011	25,343	27.3%	37.5%	10.2%

IQR, interquintile range

## Discussion

### NHS data discussion

There are some known limitations with the NHS data. Specifically, there are potential biases with both the self-reporting of physical attributes and the food recall studies used to estimate nutrient content. Various studies have shown that self-reported height and weight are systematically biased toward participants reporting they are taller and thinner than they actually are [16]. Even researchers from NHS have noted that, on average, self-reported weight was 1.0 kg less than technician-measured weight. Note that depending on actual height and weight, a 1.0 kg error in weight could change BMI by as much as 0.4 units, and a 1-inch (2.54-cm) error in height could change BMI by as much as 1.0 units. Combined, simultaneous measurement errors in weight of 1.0 kg and height of 1 inch (2.54 cm) could change BMI by almost 2.0 units relative to actual BMI [17].

The validity of food intake data are also vulnerable to systematic error or bias because of reporting inaccuracy. The relative validity of food intake estimates using a food frequency questionnaire is associated with sex, age, and other personal characteristics [18,19]. In our analysis, we used the data as they were reported and did not consider the validity of the data collection methodologies.

Due to the potential limitations and biases in the NHS datasets, we caution against focusing too heavily on the values of the point estimates from our analysis; instead, we focus on the trends and associations shown with the data, with a special emphasis on the trends in compliance with U.S. Government Nutrition Advice and changes in BMI means, medians, and distributions. As discussed further below, these trends are consistent with other published data.

### U.S. Government Nutrition Advice discussion

This section provides a brief synopsis of the history of the U.S. Government Nutrition Advice we studied and a discussion on the advisory metrics we used as proxies for overall compliance in our analysis. In 1968, the U.S. Senate Select Committee on Nutrition and Human Needs was formed, culminating in the 1977 Senate Reports [1,2]. The 1977 Senate Reports were designed to issue dietary guidelines to combat risk factors in the American diet. Specifically, the First Edition of the 1977 Senate Reports stated: “The purpose of this report is to point out that the eating patterns of this century represent as critical a public health concern as any now before us,” arguing that “too much fat, too much sugar or salt, can be and are linked directly to heart disease, cancer, obesity, and stroke, among other killer diseases. In all, 6 of the 10 leading causes of death in the U.S. have been linked to our diets.” [1]

The Second Edition of the 1977 Senate Reports, built on the First Edition but based on industry and political debate spurred in the interim, added more discussion and even a specific goal on addressing obesity, which was considered “a major risk factor in many killer diseases.” The Second Edition went on to say that obesity was “associated with the onset and clinical progression of diseases, such as hypertension, diabetes mellitus, heart disease, and gall bladder disease” and “may also modify the quality of one’s life.” The theory expounded in the Second Edition of the 1977 Senate Reports was that there was an alarming dietary energy imbalance: Americans were consuming too many calories and not burning enough of them, with obesity resulting from this energy imbalance. The solution proposed was to lower total caloric consumption by shifting from “the most concentrated energy source,” fat, to bulkier, less energy-dense, complex carbohydrate-rich foods. [2]

The 1977 Senate Reports were the predecessors to the USDGAs, issued jointly by the U.S. Department of Agriculture and the U.S. Department of Health and Human Services beginning in 1980. Thereafter, new USDGAs were issued by statute every 5 y. In it, they largely echoed the goals and recommendations of the 1977 Senate Reports for Americans to switch dietary intake from fat to complex carbohydrates and to lower saturated fat, cholesterol, sugar, and salt consumption. [1,2]. In our analysis, we examined whether the female nurses in our sample adhered to the U.S. Government Nutrition Advice on Fat Proportion, Saturated Fat Proportion, and cholesterol consumption. We isolated these 3 benchmarks because they were the only relatively consistent quantitative recommendations that were found in the guidelines starting from 1977 through to the end of our study period. We used compliance with these 3 metrics as a proxy for overall compliance with U.S. Government Nutrition Advice. We did not include the salt and sugar recommendations in our proxy for overall compliance for reasons discussed next.

Salt recommendations, which varied even in the 2 editions of the 1977 Senate Reports [1,2], were not adopted by the USDGAs until 2000, and then at different levels than the 1977 Senate Reports recommended. In addition, the reliability of sodium information with dietary recall studies is suspect, as discussed and shown in Supplemental Figure 4 [19]. Based on this, we excluded compliance with sodium recommendations to assess overall compliance status in our analysis.

Sugar recommendations also varied in the 2 editions of the 1977 Senate Reports, from a limit of no > 15% of total calories in the First Edition to no >10% of total calories in the Second Edition [1,2]. Notably, although qualitative statements on sugar consumption persisted in the USDGAs from 1980 through 2000, the first U.S. Government Nutrition Advice in the USDGAs to limit sugar consumption did not appear until 2005, which recommended a cap of only added sugar that varied based on an individual’s total fat consumption, and that was not consistent with the 1977 Senate Reports’ recommendations[[1], [2], [3], [4], [5], [6], [7], [8], [9]]. The quantitative guidance on sugar consumption in the USDGAs was dropped in 2010 [9]. Because of the lack of consistent, quantitative U.S. Government Nutrition Advice on sugar intake, we also excluded compliance with sugar recommendations when determining overall compliance status in our analysis.

### Key NHS consumption trends and U.S. Government Nutrition Advice compliance across the Studies

As shown in Figure 1, compliance with the U.S. Government Nutrition Advice on Fat Proportion, Saturated Fat Proportion, and cholesterol consumption was widespread by both the NSHI and NSHII cohorts by the 1990s. Although few respondents in our samples adhered to these collective nutritional guidelines in 1980 (only 4% of the NHSI cohort was in compliance with all 3 guidelines), a much larger share of respondents adhered to all 3 guidelines by the 1990s, with about half the sample complying overall with the guidelines in 1998. The relatively low compliance rate in 1980 is likely explained by the fact that American female nurses’ average baseline diet before the 1977 Senate Reports likely deviated substantially from the overall compliance levels in the U.S. Government Nutrition Advice [20]. Notably, however, although overall compliance peaked in the middle of the study period, the final level of overall compliance included in our analysis in 2010/2011 was just under 30% for both NHSI and NHSII, a substantial increase from the 4% overall compliance level observed in 1980.

Note that in Figure 1, the 99% CIs on the data are barely discernable until the later years. This is because the sheer number of observations, especially in the early years, means the standard error is relatively low, and the 99% CI range is extremely tight. These change in later years as survey participants drop or age out. This is also true of FIGURE 2, FIGURE 3, which also show the 99% CIs for the data from the Studies.

#### Compliance with nutrition advice on Fat Proportion

Figure 1D shows the share of each Study population complying with U.S. Government Nutrition Advice to limit Fat Proportion to <30% in any given year. In 1980, 3 years after the 1977 Senate Reports and coincident with the issuance of the first USDGA, which stated Americans should “avoid too much fat,” mean Fat Proportion was 42% of energy intake. Compliance with the 30% Fat Proportion standard was at 12%. As the U.S. Government Nutrition Advice persisted, many respondents shifted their consumption of fat in accordance with the Fat Proportion recommendation, with >50% of respondents complying with the guidelines throughout much of the 1990s. Mean Fat Proportion increased from 1990 levels in the 2000s, leading to lower compliance levels, which dropped to between 30% and 33% at the end of the Studies in 2010/11.

#### Compliance with nutrition advice on Saturated Fat Proportion

Figure 1E shows that after the 1977 Senate Reports recommended limiting Saturated Fat Proportion to <10% of total calories, and the first USDGA was issued in 1980, which stated that Americans should “avoid too much saturated fat,” consumption trended toward the U.S. Government Nutrition Advice. In 1980, mean Saturated Fat Proportion was 16%, but by 1990, it had dropped to just <11%. There was a slight uptick in mean Saturated Fat consumption for the NHSI cohort in the early 2000s, whereas the NHSII cohort slightly decreased Saturated Fat Proportion in the early 2000s. By the end of the sample period, mean compliance with saturated fat guidelines was well above mean compliance with guidelines on total fat consumption.

#### Compliance with nutrition advice on cholesterol consumption

Figure 1F shows that the U.S. Government Nutrition Advice on dietary cholesterol consumption to limit cholesterol intake to <300 mg/d was largely followed. The 1977 Senate Reports cited mean daily cholesterol consumption as high as 600 mg/d [1,2], but the first nutrition data from NHSI in 1980 showed mean consumption of 327mg/d, only slightly over the 1977 Senate Reports’ recommended limits in 1980. In the last 20 y of the sample period, ∼80%–90% of respondents complied with the cholesterol guidelines. In other words, the vast majority of female nurses followed the U.S. Government Nutrition Advice on cholesterol consumption shortly after the U.S. Government began issuing recommendations.

#### Overall compliance with government nutrition advice

Overall compliance tracked the compliance levels of Fat Proportion compliance shown in Figure 1D, just at a slightly lower level. This is because recommendations on cholesterol were adopted quickly, and compliance was high. Additionally, the Saturated Fat Proportion compliance trends were similar to but higher than those of the Fat Proportion compliance trends, which suggest that compliance with the Fat Proportion and Saturated Fat Proportion recommendations are interrelated. Because compliance with the recommended Fat Proportion was the main driver of overall compliance, we pay special attention to Fat Proportion and the prevalence of obesity when interpreting our results.

### BMI trends

Concurrent with the Study period and the introduction of U.S. Government Nutrition Advice, the mean and median BMI of female nurses in our samples increased substantially from 1980 to 2010/2011 (Figure 3). Notably, the difference between the median and mean BMI was smaller in 1980 than in 2010/2011; this is reflective of the shift in the distribution of BMI toward greater obesity levels in 2010/2011 compared with 1980. These findings are consistent with other studies that have found similar trends in the U.S. during this period, with 1 reporting the change in BMI for high-income women in North America was 1.2 per decade [21]. However, the trends shown in Figure 3 do not control for age; it is reasonable to expect that increases in age are associated with increases in BMI [21]. As such, we examined the distribution of BMI for an age-controlled sample of female nurses in 1980 compared with 2010/2011 in Figure 4, which shows that the distribution of BMI shifted such that there was an increasing share of female nurses aged 55–64 y with higher BMI in 2010/2011 compared with that in 1980.

Notably, this shift in the means, medians, and distribution of BMI persists regardless of whether respondents demonstrated overall compliance with the U.S. Government Nutrition Advice (as shown in Figure 5, which also controls for age). Both the overall compliers and overall noncompliers saw similar increases over the Study period in mean and median BMI and large increases in the share of respondents who were classified as obese or severely obese (as seen in Table 5). This is also consistent with other research that observed a similar disproportionate increase in obesity and severe obesity relative to changes in the prevalence of overweight within the period [22].

However, the data do show that there is some mitigation for the increase in BMI of overall compliers compared with overall noncompliers. Both groups saw substantial increases in mean and median BMI over the period, but the increase for overall compliers was slightly lower than that for overall noncompliers (as seen in Table 4). It is important to note though that overall compliers may have also been pursuing other activities or nutritional habits that contributed to their lower BMI, which might not have been tracked within the NHS datasets. Although some potential factors associated with a healthy lifestyle such as physical activity and smoking status were tracked in the NHS datasets, they were not comprehensively and consistently tracked over time. Accordingly, we did not control for these or other potential factors that may explain why compliers saw a smaller increase in BMI over the sample period. At best we can say that compliance was associated with a slightly lower increase in BMI than noncompliance, but future research is necessary to control for possible confounders to examine definitive causative links between compliance with U.S. Government Nutrition Advice and improved BMI outcomes. That said, had the U.S. Government Nutrition Advice been effective in mitigating obesity, we would have expected the distributional changes in BMI for compliers to have been substantially different from the distributional changes in BMI for noncompliers over the 30-y Study period. As discussed, this is not the case.

### Relationship between overall compliance and BMI

Figure 5 and Table 4 show that in 1980, there was already a difference in the BMI distributions of overall compliers compared with overall noncompliers. Noting that only a small portion of the NHSI population were overall compliers, the mean and median for overall compliers were slightly lower than those for overall noncompliers. This implies that even before the U.S. Government Nutrition Advice was issued, the subsequent overall compliers might have been engaged in lifestyle or activities that we did not consider as part of our analysis and that may or may not have been measured within the Studies, which contributed to their lower mean and median BMI.

By 2010/2011, both distributions had morphed in similar ways, and the differences in means and medians for overall compliers compared with overall noncompliers grew only slightly from those observed in 1980. As our statistical tests showed, whereas the BMI growth experienced by compliers was statistically significantly different from that of noncompliers, the magnitude of this difference was small, considering the overall upward trend in BMI over time regardless of compliance status. In fact, the impact of time on BMI growth had drastically more variation associated with it (F-value of 2696) than overall compliance (F-value of 89), and as discussed above, even that relatively small impact observed for compliance is potentially confounded by associated factors that we were not able to control for within the data.

The results shown in Table 5 show there is no definitive evidence that overall compliance led to better BMI outcomes, especially for changes in the share of those classified as obese or severely obese. Prevalance in 1980 of those with BMI of >30, and thus categorized as either obese or severely obese, were lower for overall compliers compared with overall noncompliers, 7.4% (6.5% obese plus 0.9% severely obese) compared with 12.5% (11.5% obese plus 1.0% severely obese). Over the ensuing 30 years, both groups experienced substantial growth in those with BMI >30: 23.4% of compliers (20.6% obese plus 2.8% severely obese) had BMI of >30 compared with 31.4% of noncompliers (26.1% obese plus 5.3% severely obese). However, the rate of growth for overall compliers was actually higher than that of overall noncompliers, 316% compared with 251%, respectively. The interaction between the time trends dominates compliance status here as well, as both groups more than doubled their share of obese and severely obese participants over the study period. Thus, we observed no evidence that indicates that overall compliance with U.S. Government Nutrition Advice had a meaningful mitigating effect on the larger growth trends in obesity and severe obesity from 1980 to 2010/11.

### Relationship between Fat Proportion and BMI

U.S. Government Nutrition Advice to curb fat consumption in order to restore energy balance and mitigate the prevalence of obesity [[1], [2], [3], [4], [5], [6], [7], [8], [9]], as well as the observation above that compliance with Fat Proportion advice, was the dominant driver of overall compliance, prompted us to investigate whether there was a clear, linear relationship between fat consumption and BMI. Notably, Figure 6 (and Supplemental Figure 1) shows that there is no linear relationship between Fat Proportion and BMI. Instead, the relationship between Fat Proportion and BMI appears to be conical, such that at lower BMI levels, the range of Fat Proportion is wider than the range for higher BMI levels.

The movement of Fat Proportion across survey years within each BMI category mirrored the overall shift in dietary patterns away from fat (as seen in Figure 1A and Supplemental Table 2, which shows the IQRs for all BMI categories across all years). For normal BMI, that range shifted substantially. This is best illustrated by comparing the NHSI data that started when Fat Proportion was at its maximum consumption level for the Studies in 1980 to when Fat Proportion was at its minimum consumption level. At the outset in 1980, the IQR for Fat Proportion for those with normal BMI was 32.3%–45.3%. This shows that in 1980, >80% of the sample consumed above the 30% Fat Proportion recommendation and were still able to maintain a normal BMI. Also of note is that within each survey year, the IQRs for Fat Proportion among all BMI categories are similar (as seen in Supplemental Table 3), meaning that the range of Fat Proportion was not indicative of concurrently reported BMI levels. If limiting total fat consumption were protective against BMI increases into obesity and severe obesity, we would have expected those with normal BMI to have a lower range of Fat Proportion than those categorized as obese or severely obese. This was not the case. This calls into question whether there is a meaningful relationship between individual Fat Proportion levels and BMI and obesity outcomes.

## Conclusions

Using data from the Nurses Health Studies, this paper analyzed compliance with U.S. Government Nutrition Advice from 1980 to 2011, and the concurrent trends in obesity levels and BMI distributions. American female nurses in the sample, on average, shifted their behavior toward U.S. Government Nutrition Advice on consumption of total fat and saturated fat as a share of total calories and absolute consumption of cholesterol, with overall compliance with all 3 recommendations simultaneously increasing from 4% in 1980 to a maximum approaching 60% in 1998. Nonetheless, mean and median BMI for both overall compliers and overall noncomplier increased throughout the sample period, with obesity and severe obesity growing disproportionately even as overall compliance remained widespread. We find that the distributions of BMI shifted such that a larger share of both overall compliers and overall noncompliers became obese and severely obese. We also present data that show no apparent linear association between the Fat Proportion in the diet and BMI, further throwing into doubt the idea that compliance was protective against BMI growth and obesity outcomes. The increases in BMI for female nurses in the Studies are robust regardless of compliance status. Thus, any mitigating effects compliance with U.S. Government Nutrition Advice may have had were dominated by the overall time trend. Further, those potential effects of compliance require additional research to establish whether compliance caused the minimal levels of mitigation observed, as opposed to potential confounders that were not available for control within the data.

## Data Availability

NHS I and NHS II data are maintained by the Harvard T.H. Chan School of Public Health and require their consent to access the data or analysis. The underlying data and all of our work are available via the Harvard/NHS database to any researcher with access to the data. For others, the Chan School has a process to petition or apply for access to the data.
