# Supplementary material for: Compliance with U.S. Government Nutrition Advice and Concurrent Obesity Trends Using Nurses' Health Study Data, 1980–2011
Source: J Nutr. 2023 Nov 19;154(4):1356–67. doi: 10.1016/j.tjnut.2023.11.010 (PMC11347849; doi:10.1016/j.tjnut.2023.11.010)
Supplement: Multimedia component 1 [file mmc1.docx]

# TWO-WAY ANVOA Results^[[1]](#footnote-2)^

In order to test the associations between the passage of time and compliance with U.S. Government Nutrition Advice, we estimated a two-way ANOVA model. As shown in the test statistic in Table 3 below (the “F Value” column), the largest and most consistent difference between the means of compliers’ and non-compliers’ BMIs across time is attributed to the time period. Compliance behavior, regardless of time period, also has a statistically significant association with the difference in mean BMIs. Finally, the interaction between compliance and time period is statistically significant at the 0.01 level, but the magnitude of the test statistic is much lower.

|  | **Df** | **Sum Sq** | **Mean Sq** | **F Value** | **Pr(>F)** |
| --- | --- | --- | --- | --- | --- |
| **Compliance** | 1 | 3.59 | 3.59 | 89.31 | 3.49x10^-21^** |
| **Time Period** | 1 | 108.30 | 108.30 | 2,695.58 | 0.00*** |
| **Compliance/Time Period Interaction** | 1 | 0.25 | 0.25 | 6.15 | 0.01* |
| **Residuals** | 59,413 | 2,386.96 | 0.04 |  |  |

Table A1: Two-Way ANOVA Results

Note: *** indicates a p-value approximately equal to zero; ** indicates a p-value < 0.001; * represents a p-value < 0.01.

# Supplementary BMI CLOUDS YEAR AND SURVEY


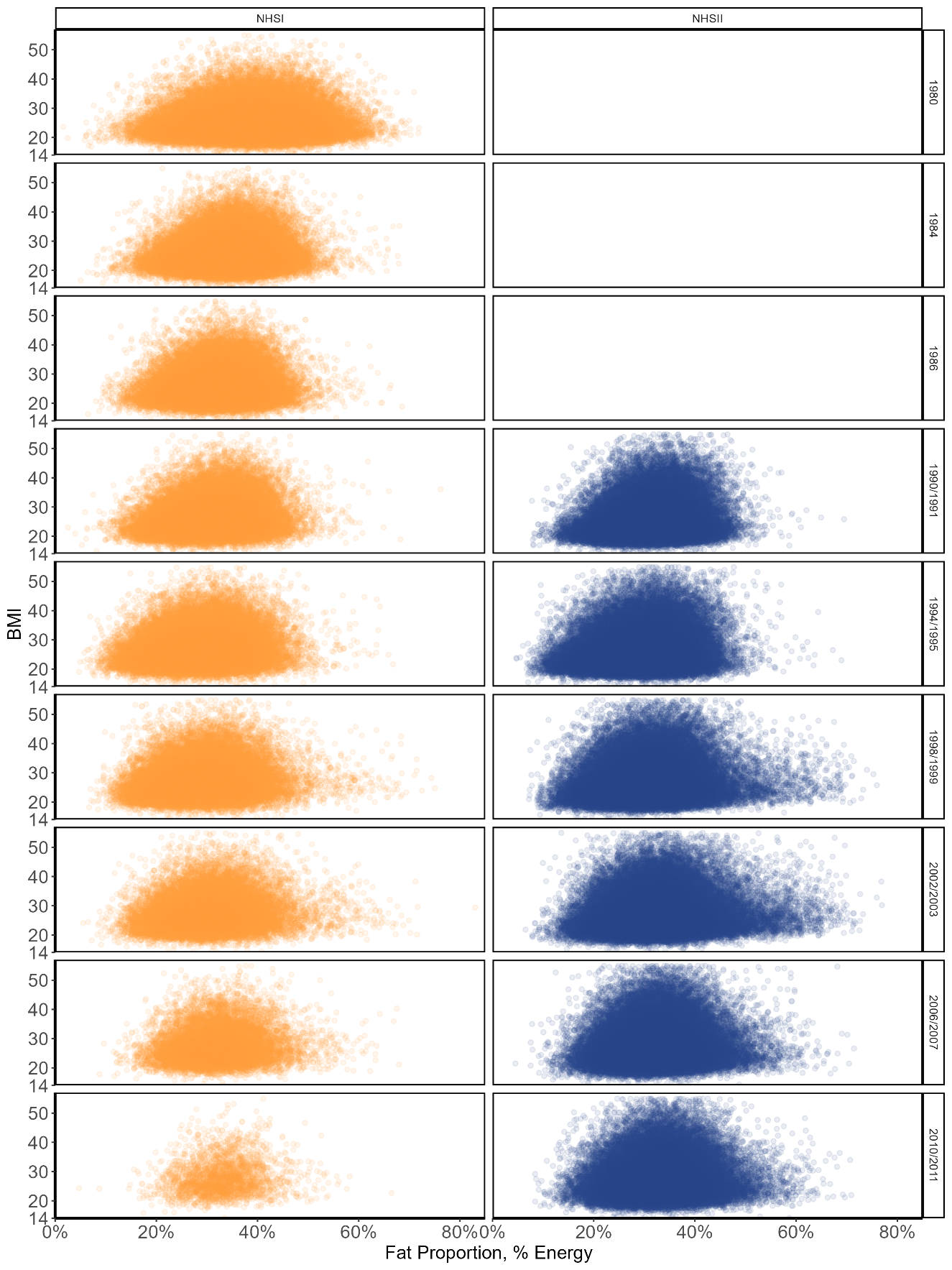


Figure A1: All Survey Years BMI vs. Fat Proportion (NHSI and NHSII)


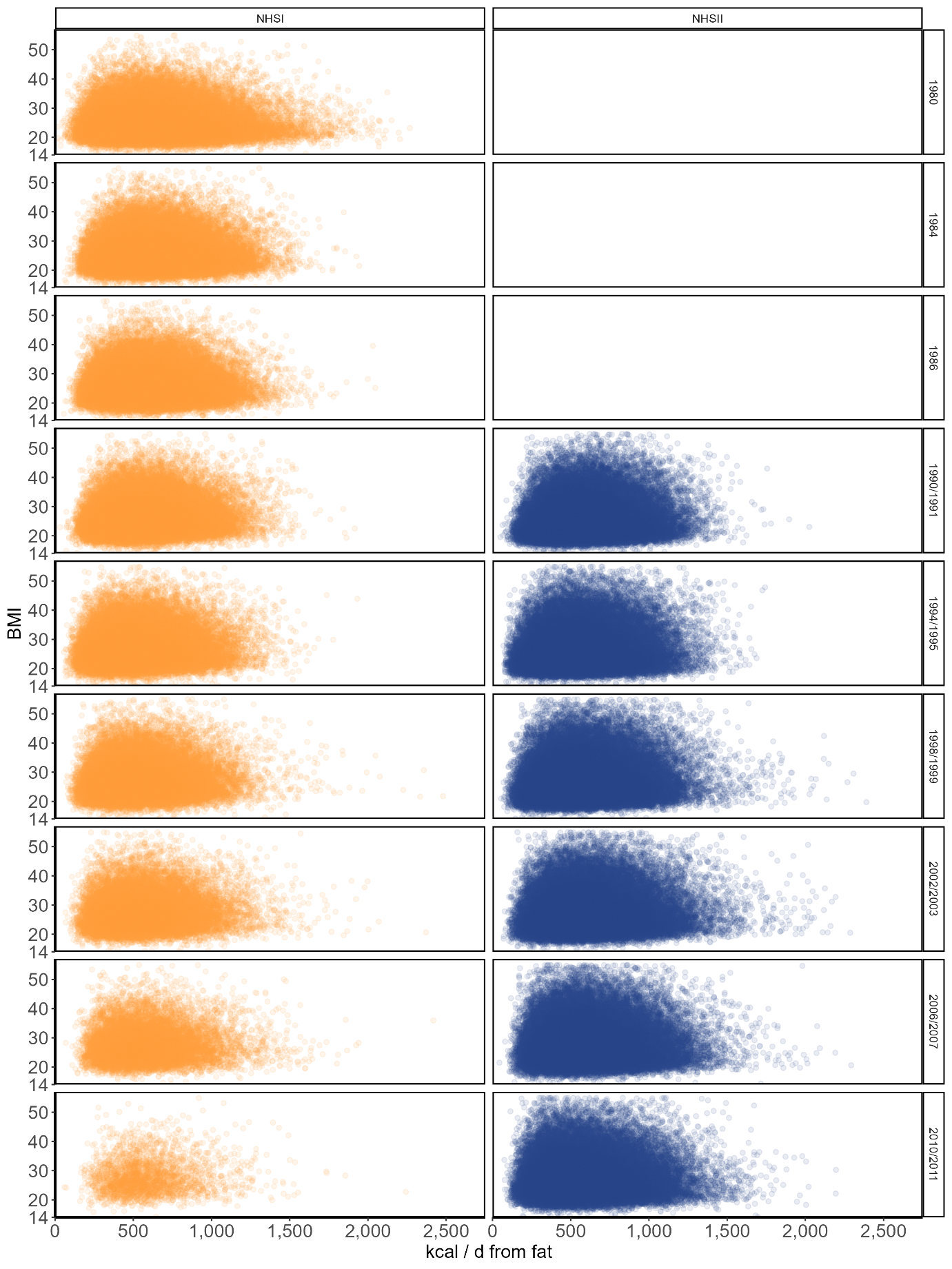


Figure A2: All Survey Years BMI vs. Calories from Fat (NHSI and NHSII)


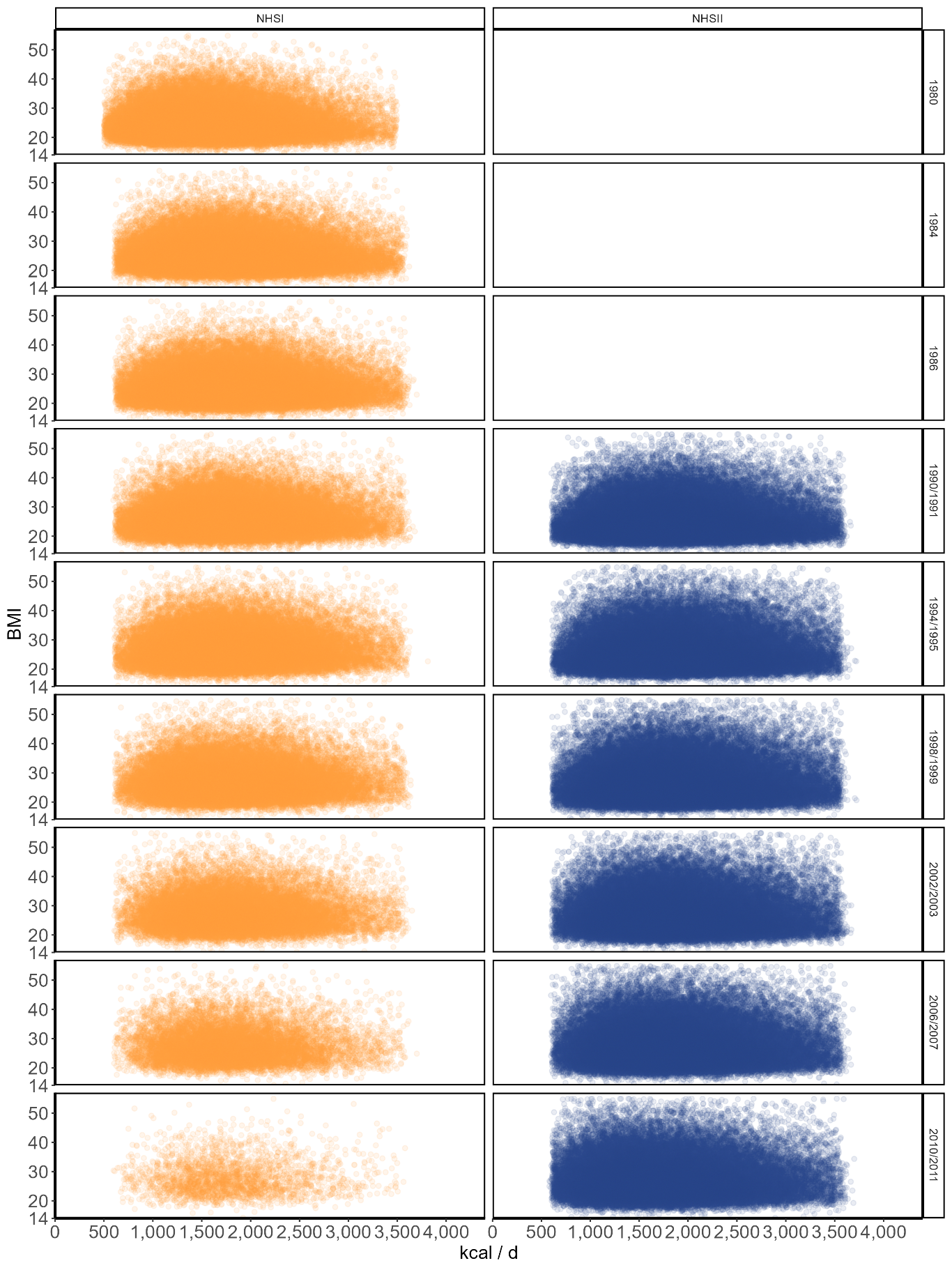


Figure A3: All Survey Years BMI vs. Daily Caloric Intake (NHSI and NHSII)

# SODIUM MEANS & MEDIANS BY SURVEY AND YEAR

As discussed in the body of the manuscript, sodium measurements in food recall surveys are generally considered unreliable. This was corroborated by the NHS data itself, which showed dubious movements in average salt consumption from 1980-1990, with average salt consumption ping-ponging from less than 1,300 mg/d in 1980 to over 3,000 mg/d in 1986, and then to more stable levels in 1990 and beyond, as shown in the Figure A4 below:


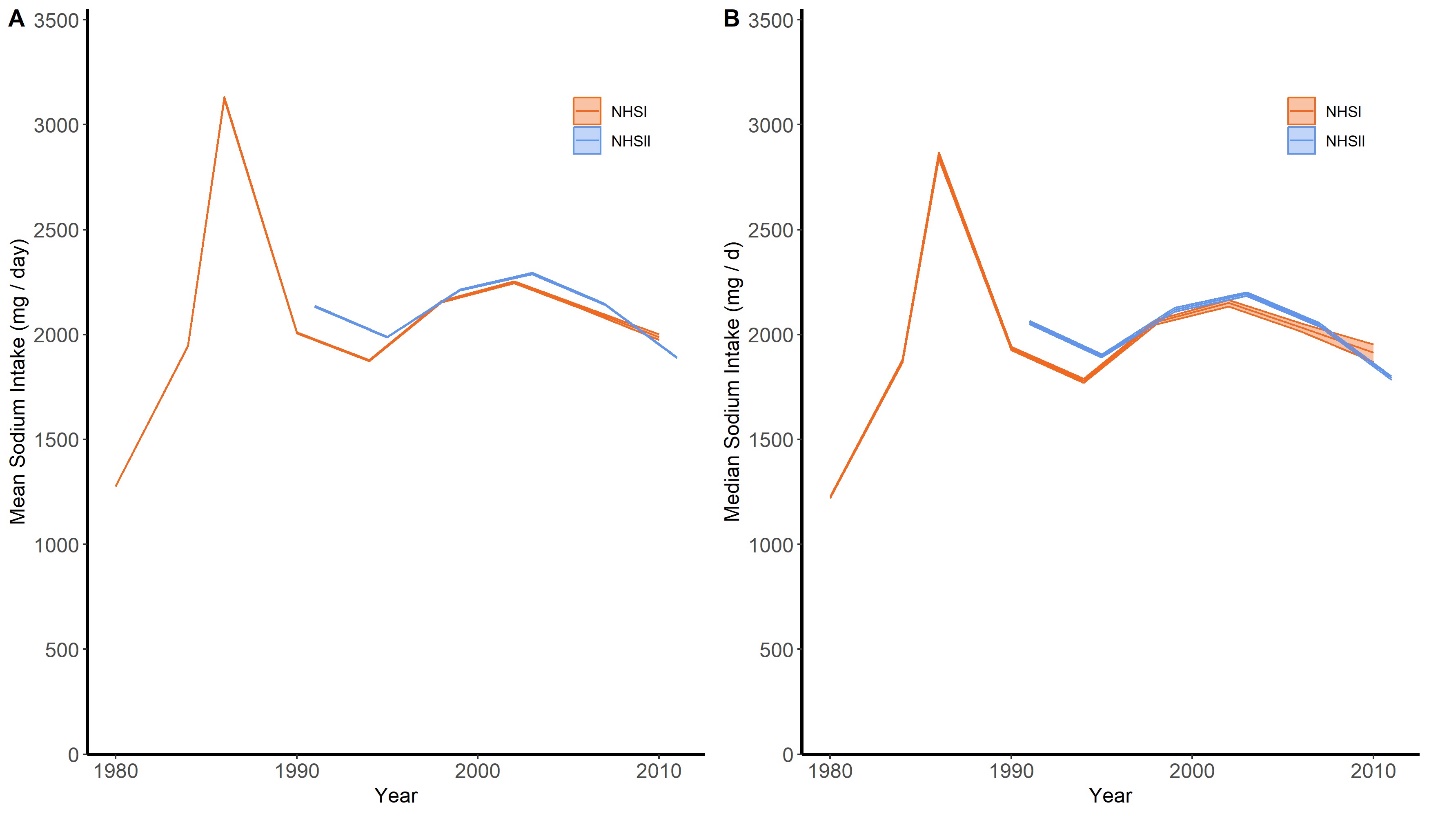


Figure A4: Mean and Median Sodium Intake for NHSI and NHSII

Shading in all panels represents 99% confidence interval for each statistic. Panel A shows mean sodium intake (in terms of mg /d) while Panel B shows median sodium intake (also in terms of mg /d).

# NHSI & NHSII Distribution Statistics

|  | **Year** | **Quintile 1** | **Mean** | **Median** | **Quintile 4** | **Interquintile Range** | **Observations** | **Standard Deviation** |
| --- | --- | --- | --- | --- | --- | --- | --- | --- |
| **NHSI** | 1980 | 32.4% | 38.9% | 39.1% | 45.4% | 13.0% | 91,472 | 7.9% |
|  | 1984 | 29.3% | 34.0% | 34.1% | 38.7% | 9.4% | 77,601 | 5.8% |
|  | 1986 | 27.4% | 32.0% | 32.0% | 36.6% | 9.2% | 70,411 | 5.7% |
|  | 1990 | 26.0% | 30.9% | 30.9% | 35.8% | 9.8% | 63,738 | 6.0% |
|  | 1994 | 23.0% | 28.6% | 28.5% | 34.1% | 11.1% | 56,321 | 6.6% |
|  | 1998 | 23.3% | 29.0% | 28.7% | 34.3% | 11.0% | 42,347 | 6.8% |
|  | 2002 | 24.7% | 30.5% | 30.1% | 35.9% | 11.2% | 25,201 | 7.2% |
|  | 2006 | 26.9% | 32.3% | 32.0% | 37.4% | 10.5% | 12,723 | 6.7% |
|  | 2010 | 28.0% | 33.1% | 33.0% | 38.0% | 10.0% | 2,586 | 6.2% |
|  |  |  |  |  |  |  |  |  |
| **NHSII** | 1991 | 26.4% | 31.0% | 31.0% | 35.6% | 9.2% | 92,562 | 5.7% |
|  | 1995 | 23.5% | 29.0% | 29.1% | 34.4% | 10.8% | 81,645 | 6.5% |
|  | 1999 | 24.8% | 30.3% | 30.0% | 35.4% | 10.6% | 81,097 | 6.9% |
|  | 2003 | 26.2% | 32.1% | 31.5% | 37.3% | 11.1% | 68,672 | 7.5% |
|  | 2007 | 27.0% | 32.0% | 31.8% | 36.8% | 9.8% | 71,286 | 6.3% |
|  | 2011 | 27.7% | 32.9% | 32.6% | 37.8% | 10.0% | 63,006 | 6.5% |

Table A2: Fat Proportion Statistics by Survey and Year (All BMI Categories)

|  | **Year** | **Quintile 1** | **Mean** | **Median** | **Quintile 4** | **Interquintile Range** | **Observations** | **Standard Deviation** |
| --- | --- | --- | --- | --- | --- | --- | --- | --- |
| **Underweight** | | | | | | | | |
| **NHSI** | 1980 | 32.0% | 38.7% | 39.1% | 45.3% | 13.3% | 1,940 | 8.1% |
|  | 1984 | 28.4% | 33.3% | 33.6% | 38.1% | 9.7% | 1,325 | 6.1% |
|  | 1986 | 26.9% | 31.7% | 32.0% | 36.9% | 10.0% | 1,133 | 6.4% |
|  | 1990 | 24.5% | 29.9% | 29.8% | 35.6% | 11.0% | 871 | 6.9% |
|  | 1994 | 21.9% | 28.2% | 28.2% | 34.2% | 12.3% | 637 | 7.5% |
|  | 1998 | 22.9% | 29.2% | 28.9% | 35.3% | 12.4% | 448 | 7.7% |
|  | 2002 | 23.1% | 29.3% | 29.1% | 36.0% | 12.9% | 265 | 7.9% |
|  | 2006 | 25.5% | 30.6% | 30.8% | 36.5% | 10.9% | 137 | 6.9% |
|  | 2010 | 24.3% | 29.5% | 29.7% | 33.6% | 9.3% | 25 | 6.1% |
|  |  |  |  |  |  |  |  |  |
| **NHSII** | 1991 | 25.0% | 29.7% | 29.9% | 34.4% | 9.4% | 2,549 | 6.0% |
|  | 1995 | 22.6% | 28.6% | 28.7% | 34.5% | 11.9% | 1,573 | 7.0% |
|  | 1999 | 22.4% | 28.6% | 28.7% | 34.5% | 12.1% | 1,181 | 7.2% |
|  | 2003 | 24.4% | 30.0% | 30.1% | 35.4% | 11.0% | 805 | 7.1% |
|  | 2007 | 24.7% | 30.4% | 30.5% | 36.1% | 11.4% | 800 | 7.2% |
|  | 2011 | 25.6% | 31.6% | 31.4% | 37.0% | 11.3% | 770 | 7.1% |
|  | **Year** | **Quintile 1** | **Mean** | **Median** | **Quintile 4** | **Interquintile Range** | **Observations** | **Standard Deviation** |
| **Overweight** | | | | | | | | |
| **NHSI** | 1980 | 32.4% | 39.0% | 39.2% | 45.6% | 13.1% | 21,177 | 7.9% |
|  | 1984 | 29.6% | 34.2% | 34.2% | 38.9% | 9.3% | 20,746 | 5.7% |
|  | 1986 | 27.7% | 32.2% | 32.2% | 36.7% | 8.9% | 19,984 | 5.5% |
|  | 1990 | 26.4% | 31.2% | 31.2% | 35.9% | 9.5% | 19,427 | 5.8% |
|  | 1994 | 23.2% | 28.6% | 28.5% | 34.0% | 10.7% | 18,528 | 6.5% |
|  | 1998 | 23.5% | 29.0% | 28.6% | 34.1% | 10.6% | 14,509 | 6.8% |
|  | 2002 | 24.9% | 30.5% | 30.0% | 35.7% | 10.8% | 8,609 | 7.1% |
|  | 2006 | 26.8% | 32.2% | 31.8% | 37.4% | 10.6% | 4,332 | 6.7% |
|  | 2010 | 27.8% | 33.1% | 32.8% | 37.6% | 9.8% | 895 | 6.3% |
|  |  |  |  |  |  |  |  |  |
| **NHSII** | 1991 | 27.1% | 31.6% | 31.5% | 36.0% | 8.9% | 19,168 | 5.5% |
|  | 1995 | 23.8% | 29.1% | 29.1% | 34.4% | 10.6% | 19,970 | 6.3% |
|  | 1999 | 25.0% | 30.5% | 30.1% | 35.5% | 10.5% | 21,451 | 7.0% |
|  | 2003 | 26.3% | 32.3% | 31.5% | 37.4% | 11.0% | 19,171 | 7.6% |
|  | 2007 | 27.0% | 32.0% | 31.7% | 36.7% | 9.6% | 21,165 | 6.2% |
|  | 2011 | 27.6% | 32.8% | 32.5% | 37.6% | 10.0% | 18,973 | 6.4% |
|  | **Year** | **Quintile 1** | **Mean** | **Median** | **Quintile 4** | **Interquintile Range** | **Observations** | **Standard Deviation** |
| **Obese** | | | | | | | | |
| **NHSI** | 1980 | 33.1% | 39.5% | 39.6% | 46.1% | 13.1% | 9,149 | 7.8% |
|  | 1984 | 30.6% | 35.2% | 35.2% | 39.8% | 9.2% | 9,414 | 5.8% |
|  | 1986 | 28.4% | 33.0% | 33.1% | 37.6% | 9.1% | 9,243 | 5.6% |
|  | 1990 | 27.4% | 32.3% | 32.3% | 37.0% | 9.6% | 9,509 | 5.9% |
|  | 1994 | 24.6% | 30.0% | 29.9% | 35.3% | 10.8% | 10,952 | 6.5% |
|  | 1998 | 24.6% | 30.1% | 29.8% | 35.1% | 10.5% | 9,068 | 6.6% |
|  | 2002 | 26.0% | 31.7% | 31.2% | 36.9% | 10.9% | 5,928 | 7.0% |
|  | 2006 | 28.0% | 33.2% | 32.9% | 38.1% | 10.1% | 3,162 | 6.4% |
|  | 2010 | 28.9% | 33.9% | 33.9% | 38.5% | 9.5% | 678 | 5.7% |
|  |  |  |  |  |  |  |  |  |
| **NHSII** | 1991 | 28.0% | 32.5% | 32.6% | 37.0% | 9.0% | 10,778 | 5.5% |
|  | 1995 | 25.1% | 30.4% | 30.5% | 35.5% | 10.4% | 12,797 | 6.3% |
|  | 1999 | 26.2% | 31.6% | 31.2% | 36.6% | 10.4% | 14,873 | 6.9% |
|  | 2003 | 27.2% | 33.1% | 32.6% | 38.3% | 11.1% | 14,128 | 7.4% |
|  | 2007 | 27.8% | 32.7% | 32.4% | 37.4% | 9.6% | 16,279 | 6.1% |
|  | 2011 | 28.3% | 33.4% | 33.2% | 38.1% | 9.8% | 14,944 | 6.3% |
|  | **Year** | **Quintile 1** | **Mean** | **Median** | **Quintile 4** | **Interquintile Range** | **Observations** | **Standard Deviation** |
| **Severely Obese** | | | | | | | | |
| **NHSI** | 1980 | 32.7% | 39.7% | 39.7% | 46.7% | 14.0% | 845 | 8.0% |
|  | 1984 | 31.1% | 35.9% | 35.9% | 40.6% | 9.4% | 1,020 | 6.0% |
|  | 1986 | 29.2% | 33.8% | 33.9% | 38.6% | 9.4% | 1,006 | 6.0% |
|  | 1990 | 28.7% | 33.7% | 33.8% | 38.3% | 9.6% | 1,085 | 5.9% |
|  | 1994 | 25.7% | 31.2% | 31.4% | 36.3% | 10.5% | 1,382 | 6.6% |
|  | 1998 | 26.2% | 31.5% | 31.4% | 36.5% | 10.3% | 1,278 | 6.8% |
|  | 2002 | 27.1% | 32.7% | 32.4% | 38.0% | 10.9% | 899 | 6.7% |
|  | 2006 | 29.0% | 33.9% | 33.4% | 38.5% | 9.5% | 492 | 6.1% |
|  | 2010 | 29.7% | 34.9% | 35.0% | 39.7% | 10.0% | 109 | 5.4% |
|  |  |  |  |  |  |  |  |  |
| **NHSII** | 1991 | 28.9% | 33.9% | 33.9% | 38.7% | 9.8% | 1,908 | 5.9% |
|  | 1995 | 26.5% | 32.0% | 32.1% | 37.5% | 11.0% | 2,666 | 6.6% |
|  | 1999 | 27.3% | 32.8% | 32.6% | 38.0% | 10.7% | 3,229 | 6.8% |
|  | 2003 | 28.6% | 34.4% | 34.0% | 39.3% | 10.8% | 3,099 | 7.4% |
|  | 2007 | 28.8% | 33.5% | 33.4% | 38.1% | 9.3% | 3,580 | 6.0% |
|  | 2011 | 29.3% | 34.1% | 33.9% | 38.8% | 9.5% | 2,976 | 6.0% |

Table A3: Fat Proportion Statistics by Survey, Year, and BMI Category

1. Supplementary Data includes: Table A1: Two-Way ANOVA model results testing the difference between the means of compliers’ and non-compliers’ BMIs; Figure A1: All Survey Years BMI vs. Far Proportion; Figure A2: All Survey Years BMI vs. Calories from Fat; Figure A3: All Survey Years BMI vs. Daily Caloric Intake; Figure A4: Mean and Median Sodium Intake for NHSI and NHSII; Table A2: Fat Proportion Statistics by Survey and Year (All BMI Categories); Table A3: Fat Proportion Statistics by Survey, Year and BMI Category. [↑](#footnote-ref-2)
